# Treatment of a cancer patient by an adoptive cell therapy protocol combining DC vaccination with cbl-b ex vivo silencing

**DOI:** 10.1186/2051-1426-3-S2-P172

**Published:** 2015-11-04

**Authors:** Monika Sachet, Guenther Lametschwandtner, Hubert Hayden, Michaela Hassler, Hans Loibner, Pierre Triozzi, Josef Friedl

**Affiliations:** 1 Department of Surgery, Medical University Vienna, Vienna, Austria; 2 Apeiron Biologics, Vienna, Austria; 3 Medical University of Vienna, Vienna, Austria; 4 Wake Forest University, Winston-Salem, NC, USA

## Background

The E3 ubiquitin ligase cbl-b has been identified as a key intracellular checkpoint limiting T and NK cell activation. Concordantly, blockade of cbl-b function by genetic deletion strongly enhances anti-tumor immune responses, thereby validating cbl-b as target for immunotherapy. We have recently shown in the B16-OVA model that transfer of transiently cbl-b silenced murine T cells together with DC vaccination could suppress tumor growth. This provides a rationale to combine administration of cbl-b silenced PBMCs with an established DC vaccination protocol. We present here a case study for the treatment of a patient suffering from lung metastases originating from pancreatic cancer.

## Methods

PBMCs were isolated ex vivo from the cancer patient and transfected with cbl-b siRNA by electroporation. DCs were pulsed with lysates of pancreatic tumor cell lines serving as antigen source and co-administrated with the PBMCs intranodally to the cancer patient. The composition and phenotype of the immune compartment of the patient was regularly monitored at each treatment by flow cytometry. The phenotype and activation of DCs and cbl-b silenced PBMCs was assessed by flow cytometry and ELISA. Tumor progression was monitored by determination of CA19-9 blood levels and computer tomography.

## Results

Ex vivo Transfection of cancer patient PBMCs with cbl-b si RNA resulted in strong suppression of cbl-b expression and enhanced T cell activation. No adverse events were observed when cbl-b silenced PBMCs (up to 1.5*10^8) were administrated to the patient. When cbl-b silenced PBMCs were co-administrated with activated DCs, no worsening of DC-associated slight fever and local DTH reactions was observed. Overall, 2 vaccination cycles (each 10 single treatments with DCs and cbl-b silenced PBMCs) were administrated. During the treatment, massive fluctuations in the PBMC immune compartments were observed, indicating the impact on the patient`s immune system. Tumor progression was slowed during the treatment periods, resulting in disease control over a period of more than 30 months.

## Conclusions

The adoptive cell therapy protocol combining DC vaccination and ex vivo cbl-b silencing was shown to be feasible and well tolerated. Moreover, immunologic effects and slowed tumor growth suggest beneficial effects of the treatment. Based on these results, a Phase I trial for adoptive cell therapy with larger numbers of cbl-b silenced PBMCs was started at Wake Forest University.

